# Utilization of maternity waiting homes: before, during, and after the Ebola virus disease outbreak in Bong County, Liberia

**DOI:** 10.1093/inthealth/ihz039

**Published:** 2019-07-11

**Authors:** Joseph E Perosky, Karina Z McLean, Alphonso Kofa, Aloysius Nyanplu, Michelle L Munro-Kramer, Jody R Lori

**Affiliations:** 1 Michigan State University, College of Human Medicine, 965 Fee Rd A110, East Lansing, MI, USA; 2 University of Michigan, 400 N. Ingalls Bldg. Suite 3320, Ann Arbor, MI USA; 3 Bong County Health Team, Suakoko, Liberia; 4 University of Michigan, 400 N. Ingalls Bldg. Ann Arbor, MI, USA

**Keywords:** Ebola virus disease, Liberia, maternity waiting homes

## Abstract

**Background:**

Maternity waiting homes (MWHs) are used to increase the number of women delivering at health care facilities. The first MWHs in Liberia were opened in 2012, prior to the Ebola virus disease (EVD) outbreak.

**Methods:**

Longitudinal data were collected from registries on MWH use, antenatal care, postnatal care and facility deliveries from 2012 to 2016 to assess MWH utilization.

**Results:**

All indicators examined declined during the EVD outbreak, but within 6 months of the cessation of the outbreak they returned to pre-EVD levels.

**Conclusions:**

Findings suggest MWH utilization remained stable after international funding ceased and EV affected the region.

## Introduction

Liberia is a nation with extreme needs related to maternal and newborn health. Social and health structures were devastated during 14 y of civil wars, and that was before the 2014–2015 Ebola virus disease (EVD) outbreak that further weakened an already ailing health system. The EVD outbreak decimated the country, leading to a total of 10 675 cases of EVD and resulting in 4809 deaths.^1^ As EVD spread across the country, fear of contracting the disease and a mistrust of the health care system grew.^2^ Clinicians left their posts, those in need of health care did not report to health care facilities due to fear of contracting EVD and any medical equipment was instead utilized against EVD and taken away from maternal care. Maternal health access was one of the most severely impacted sectors of health care in the countries affected by EVD.^3^

Maternity waiting homes (MWHs) are shelters built near a health facility where pregnant women can stay temporarily prior to delivery and immediately postpartum. MWHs have the potential to decrease maternal and newborn death and increase facility deliveries.^4^ However, MWHs are a relatively new intervention and, to date, no longitudinal data about their utilization have been reported. The purpose of this time series analysis is to assess how temporal use of MWHs, facility delivery, and antenatal and postnatal care changed before, during and after the EVD outbreak in rural Liberia.

## Materials and methods

In 2012, with funding from the U.S. Agency for International Development, a two-group comparison design study was conducted at remote primary health facilities (those with and without MWHs) in Bong County, Liberia to evaluate their impact on maternal and newborn outcomes. Bong County is one of the largest counties per capita in Liberia, with an estimated population of >330 000 and 37 health facilities. The MWHs were managed by a community governance committee comprised of traditional birth attendants and local residents.^4^ Results from the beginning of this study showed a decrease in maternal and perinatal mortality in communities with an MWH compared with those without one.^4^

The original five MWHs in Bong County, Liberia continued to collect data after the project ended in 2014; however, two of these MWHs were excluded from the analysis due to incomplete and missing clinic registries following the EVD outbreak. These data were assessed using a retrospective study design to gain a better understanding of longitudinal MWH use. Data were recorded in clinic registries from 2012 to 2016, extracted by Liberian research assistants and analysed using one-way analysis of variance using SPSS (IBM, Armonk, NY, USA). Residuals were normally distributed, with the exception of the pre-Ebola time period for MWH stays and facility deliveries. Alpha levels of 0.05 were used for statistical significance. We examined differences in MWH use, facility delivery, and antenatal and postnatal care before, during and after the EVD outbreak and cessation of funding support for the MWHs. Data collected include the number of women staying at the MWHs per month, number of deliveries at the facility associated with the MWH per month, number of women attending 6-week postnatal care (PNC) visits at the facility associated with the MWH, and number of women receiving four or more antenatal care (ANC) visits at the facility associated with the MWH.

## Results

**Figure 1.**
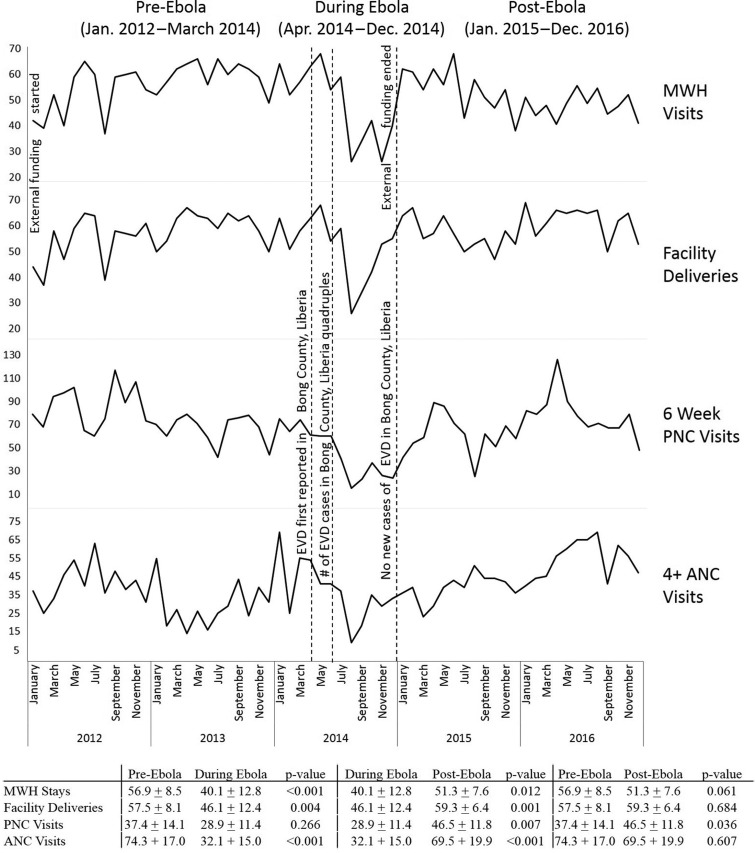
There was a decrease in the number of women staying at MWHs, deliveries taking place in a facility and ANC visits during the EVD outbreak in Bong County, Liberia compared with pre-outbreak levels. Subsequently there was an increase in MWH stays, facility deliveries, ANC visits and PNC visits post-Ebola compared with during the outbreak. PNC visits post-Ebola have increased beyond pre-Ebola levels.

MWH use increased from 2012 to 2013, prior to the EVD outbreak, with the highest number of stays occurring between January and March of 2014, which also coincided with the ramp up of study activities. The EVD outbreak was declared in Bong County in April 2014 and by June 2014 the outbreak was spreading rapidly, with cases quadrupling each month. As the outbreak continued, compared with pre-Ebola levels, the use of MWHs (p<0.001), the number of ANC visits (p<0.001), the occurrence of facility-based delivery (p <0.05) and the number of PNC visits (p = 0.266) declined, reaching their lowest point in August 2014 (Figure 1). By November 2014, the number of EVD cases in Bong County stabilized. By December 2014, there were no new cases of EVD in Bong County. At the end of 2014, as the EVD outbreak was subsiding, funding support for the project came to an end. By January 2015, MWH use (p=0.061), facility-based delivery (p=0.684) and ANC visits (p=0.607) returned to pre-EVD levels. PNC visits post-Ebola have increased beyond pre-Ebola levels (p=0.036).

## Discussion

Overall, MWHs have been found to be an integral health system intervention in Bong County, Liberia due to continued use after international funding ended and EVD affected the region. Although the use of MWHs, facility-based delivery, and antenatal and postnatal care declined at the peak of EVD, their use increased once the region became stabilized. Although no previous studies have evaluated the reasons for a decrease in MWH use during EVD, the causes likely mirror causes in the reduction in maternal health service uptake that have been previously reported.^5^ There was a trend towards a reduction in MWH use after the EVD outbreak compared with before the outbreak. This may be due to the cessation of funding support, which included maintenance of the MWH, food security, incentive for the staff at the MWH and furnishing the MWH with supplies. Interestingly, there has been an increase in PNC visits following the EVD outbreak. Importantly, no previous studies have investigated MWH use and maternal health service access during the recovery period from Ebola. Further investigation into the mechanisms and causes for a rapid 6-month return to pre-EVD levels of MWH use and maternal health services access is needed.

**Authors’ contributions:** JRL and MLM conceived the study. JRL designed the study protocol. JEP, KZM, AK and AN carried out data collection and analysis and interpretation of the data. JEP and KZM drafted the manuscript. JEP, JRL, MLM, AK and AN critically revised the manuscript for intellectual content. All authors read and approved the final manuscript. JEP and JRL are guarantors of the paper.

**Acknowledgments:** We would like to thank Dr James Barclay for overseeing the logistics of personnel for data collection.

**Funding:** This work was supported by the Bill and Melinda Gates Foundation (OPP1170983); https://www.gatesfoundation.org/How-We-Work/Quick-Links/Grants-Database/Grants/2017/06/OPP1170983. The funders had no role in study design, data collection and analysis, decision to publish or preparation of the manuscript. The content is solely the responsibility of the authors and does not necessarily reflect the positions or policies of the Bill and Melinda Gates Foundation.

**Competing interests:** None declared.

**Ethical approval:** Prior to data collection, institutional review board (IRB) approval was received from the University of Michigan Health Sciences and Behavioral Sciences IRB and the University of Liberia Pacific Institute for Research and Evaluation.
